# NEUROCOG-DysNet: a multi-scale cognitive feature engineering framework with stacking ensemble for improved dyslexia screening

**DOI:** 10.3389/frai.2026.1909141

**Published:** 2026-09-02

**Authors:** Sudha M, Murali S

**Affiliations:** 1 School of Advanced Sciences (SAS), Vellore Institute of Technology, Vellore, Tamil Nadu, India; 2 School of Computer Science and Engineering (SCOPE), Vellore Institute of Technology, Vellore, Tamil Nadu, India

**Keywords:** cognitive feature engineering, dyslexia screening, ensemble learning, gamified assessment, machine learning, stacking meta-learner

## Abstract

**Introduction:**

Dyslexia affects around 10% of the global population, yet early screening depends heavily on resource-intensive clinical assessments that are difficult to scale. Gamified cognitive platforms offer a practical alternative, but most existing machine learning approaches rely on raw task metrics without exploiting the hierarchical cognitive structure of multi-task assessment batteries.

**Methods:**

NEUROCOG-DysNet is a multi-scale cognitive feature engineering and stacking ensemble framework for dyslexia screening using gamified assessment data from the Dytective platform (*N* = 3,644; 392 dyslexic, 3,252 non-dyslexic). The framework extracts 258 hierarchical features across five cognitive levels-task, phase, temporal, global, and cross-domain-from raw game interactions, supplemented by four demographic variables. LightGBM-based feature selection retains the 50 most discriminative features. Seven base classifiers, configured to emphasize high sensitivity, balanced performance, or high AUC, are combined using five ensemble strategies and evaluated under a leakage-safe nested cross-validation protocol.

**Results:**

The stacking meta-learner achieves the highest composite score and attains an AUC-ROC of 0.8924 (95% CI: 0.8543–0.9253), sensitivity of 0.8077, specificity of 0.8187, and balanced accuracy of 0.8132. DeLong's test (*p* = 0.6946) and McNemar's test (*p* = 0.1443) indicate that AUC gains over the strongest individual baseline (XGB_HighAUC) are not statistically significant. Nested cross-validation confirms a small optimism gap (+0.011 AUC). Clinical utility analysis yields a 5.25 × lift in the highest-risk decile and a negative predictive value of 0.9726. Ablation shows that demographic variables contribute more to discrimination than any engineered cognitive level; a demographics-free variant attains AUC 0.7948.

**Discussion:**

NEUROCOG-DysNet provides a tunable sensitivity-specificity balance and well-calibrated risk scores suitable for a first-stage screening aid. The ensemble's advantage over fixed-weight alternatives is marginal on this dataset, and the cross-domain feature level is effectively inert. External validation across independent language cohorts and prospective multi-center evaluation are required before clinical deployment.

## 1 Introduction

Dyslexia is a specific learning disability of neurobiological origin, leading to persistent difficulties in correct and swift word recognition, poor spelling, and lowered decoding skill (Shaywitz, 1998; Lyon et al., 2003). Epidemiological data indicate that dyslexia affects between 5% and 17% of people worldwide, making it the most common learning disability (Peterson and Pennington, 2012). The negative effects of dyslexia, if left undiagnosed, do not only surface in poor academic performance but also include lowered self-esteem, increased anxiety, and long-lasting socioeconomic disadvantage (Livingston et al., 2018). Therefore, it is very important to recognize dyslexia in the earliest stages because early intervention can not only substantially reduce these negative effects but can also change the lives of affected children to be successful academically as well as personally (Snowling and Hulme, 2012).

Dyslexia screening is still mainly through standardized tests given by professionals trained in this field. This process requires considerable time and personnel and is difficult to scale (Zingoni et al., 2024). Standard diagnostic tests like CTOPP-2, WRMT, and GSRT, although well validated, are demanding to administer and require specialized expertise, which in turn causes waitlists in educational and healthcare systems (Ahire et al., 2023). Thus, there is a long wait from the diagnosis of dyslexia for many children, they are mostly recognized after experiencing academic failure for a few years, by which point the cumulative deficits in reading and related skills have become deeply entrenched. Also, a lack of access to trained diagnosticians in developing countries and poor communities makes this problem even worse (Rello et al., 2016, 2020).

One potential application of gamified cognitive assessments is dyslexia screening on a large scale. For example, Dytective (Rello et al., 2016, 2020) captures behavioral data such as accuracy, response patterns, and error profiles across a set of linguistically grounded game tasks, which focus on visual processing, phonological awareness, and working memory. These data streams harbor hidden diagnostic signals, which if properly analyzed, can lead to screening accuracies similar to or even better than those obtained by traditional instruments (Rauschenberger et al., 2020). Meanwhile, even though analyzing gamified data for screening through machine learning is a viable approach, most of them have only depended on basic task-level metrics without considering the deep hierarchical cognitive structure in multi-task assessment batteries. This limitation constrains discriminative power and fails to capture the cross-domain cognitive patterns that are hallmarks of dyslexia.

NEUROCOG-DysNet, a cognitively multi-level feature extraction framework combined with a stacking ensemble meta-learner, to improve dyslexia screening is presented in the paper. Firstly, the conversion of raw gamified assessment data to 258 features arranged in five hierarchical cognitive levels is done in a systematic way by the proposed method. Secondly, the LightGBM-based feature selection is then used to pick the top 50 most informative features. Lastly, seven highly diverse base classifiers are combined using a stacking architecture that is optimized. Two main shortcomings of the existing literature are the target of this framework. Firstly, the limited usage of multi-scale cognitive feature representations. Secondly, the lack of well-founded ensemble methods that not only provide a good trade-off between sensitivity and specificity but also lead to clinically relevant screening are the two aspects targeted by this work.

### 1.1 Contributions

The major contributions of this work are summarized as follows:

- The paper proposes a new multi-scale cognition feature engineering pipeline NEUROCOG-DysNet through which 258 hierarchical features across five cognitive levels (task-level, phase-level, temporal, global, and cross-domain) are extracted from gamification-based dyslexia assessment data. Besides the conventional raw accuracy metrics, these features capture finer-grained cognitive signatures. We qualify this contribution in light of our own ablation: demographic variables contribute more to discrimination than any engineered level, and the cross-domain level is effectively inert, so the hierarchy is a useful but not dominant source of signal.
- Five different ensemble strategies namely, sensitivity-weighted, AUC-weighted, optimized-weighted, two-stage cascade, and stacking meta-learner were systematically compared, with the stacking method being the best as it got an AUC-ROC of 0.8924 (95% CI: 0.8543–0.9253) and a sensitivity of 0.8077 on the test data kept separate.
- A rigorous statistical evaluation shows that the stacking ensemble attains an AUC comparable to the strongest individual baseline (the AUC difference is not significant; DeLong *p* = 0.6946), while McNemar's test indicates a change in classification decisions (*p* = 0.1443). The framework's advantage lies in a controllable sensitivity–specificity trade-off and well-calibrated risk scores rather than a higher AUC.
- This paper includes a detailed clinical utility analysis that covers different risk stratification levels, lift analysis (5.25 × at the top 10% percentile), and predictive values (NPV = 0.9726), showing the real-world usefulness of NEUROCOG-DysNet for widespread screening.

The rest of this paper is organized as follows. Section 2 provides a comprehensive review of related work and identifies research gaps. Section 3 describes the proposed NEUROCOG-DysNet framework in detail. Section 4 lays out how the experiments have been set up, such as a description of the dataset, baseline methods, and evaluation metrics. Section 5 shows and analyzes test/experimental results. Section 6 interprets the results, and their significance. Finally, Section 7 summarizes the paper and points to new paths for future research.

## 2 Related work

### 2.1 Traditional and clinical dyslexia screening

In the past, identifying dyslexia clinically has mainly relied on psychometric test batteries that assess phonological processing, rapid automatized naming, and reading fluency. Foundational characterization of dyslexia's neurobiological basis that has led to the development of subsequent assessment tools was provided by Shaywitz (1998). Standardized instruments such as the CTOPP (Comprehensive Test of Phonological Processing) and the Woodcock Reading Mastery Tests have long been considered gold standards, but their administration requires trained professionals and is time-consuming (Peterson and Pennington, 2012). Mather and Schneider (2023) discussed the role of cognitive testing in dyslexia assessment and pointed out that the phonological processing, working memory, and processing speed tests are all important in diagnosis, but they remain resource-intensive. The issue of early diagnosis and how it is closely linked to the need for starting formal reading instruction was raised by Snowling and Hulme (2012), who further observed that pre-literacy indicators such as phonological awareness and letter knowledge can be used to predict reading difficulties. Research conducted by Psyridou et al. (2021), 2023) revealed the possibility of profiling reading and math difficulties from kindergarten to middle school, thus highlighting the role of early cognitive markers. Nevertheless, the requirement for expert administration limits the scalability and population-level screening, especially in resource-poor settings (Lyon et al., 2003). Livingston et al. (2018) reported the wider psychosocial effects of delayed diagnosis, which they considered a strong argument for the development of accessible and scalable alternatives to conventional clinical assessment paradigms.

### 2.2 Gamified and technology-assisted screening approaches

The digitization of cognitive assessments has created opportunities for scaling dyslexia screening. Rello et al. (2016), 2020) have constructed the Dytective platform, a gamified online test that, through linguistically grounded tasks, predicts dyslexia risk with over 80% detection accuracy in a study with more than 3,600 participants. Expanding on this, Rauschenberger et al. (2020) looked at language-independent screening through web-games with visual and auditory cues, showing possible cross-linguistic generalization. Kaisar and Chowdhury (2022) used oversampling and ensemble machine learning to the imbalanced Dytective datasets, underlining a class imbalance problem in dyslexia screening situations. Vajs et al. (2023), in another development, used autoencoders to represent eye-tracking images for dyslexia detection and showed that visual attention during reading carries substantial discriminative information that generalizes across language boundaries. Research shows that collecting data through computer games has several benefits compared to traditional assessments: lower workload in administration, children participating because of the engagement factor, and the production of rich multi-dimensional behavioral data streams (Rello et al., 2016). However, up to now, most methods have mainly used simple game metrics such as raw accuracy and click counts, i.e. without a systematic feature engineering that represents the hierarchically organized cognitive processes that are at the basis of task performance.

### 2.3 Machine learning for dyslexia detection

The scope of machine learning methodologies in dyslexia diagnosis has diversified at a fast pace, engaging modalities such as handwriting analysis and neuroimaging. First, Ahire et al. (2023) work presents a comprehensive survey covering support vector machines, random forests, and neural network architectures across various dyslexia-related datasets. Alqahtani et al. (2023) revisited deep learning studies as potent means for dyslexia prediction and gave examples of top performance by convolutional and recurrent architectures with EEG and handwriting datasets. However, Usman et al. (2021) even before that examined machine learning-based dyslexia biomarker detection methods along with implementation challenges such as small sample sizes, heterogeneous data modalities, and the unavailability of standardized benchmarks. With regards to cognitive assessment data, Zingoni et al. (2024) built an SVM-based classification model that identified university students with dyslexia largely from self-evaluation questionnaire data. As a follow-up to this, Jan and Khan (2023) performed a systematic review of the research dimensions leading to dyslexia screening with machine learning and came up with data scarcity, feature representation, and model interpretability as the most persistent of challenges. Alkhurayyif and Sait (2024), instead, tackled the problem with a detection model that was deep learning-driven and used multi-modality data combining fMRI, MRI, and EEG. They reported high accuracy through squeeze-and-excitation networks and attention mechanisms. Gomolka et al. (2024) proposed an LSTM network that was fused with eye-tracking technology to diagnose dyslexia in early school-aged children. The network obtained promising results in temporal sequence classification from gaze patterns. Alrubaian (2025) turned to cutting-edge machine learning algorithms such as XGBoost and random forest to pinpoint major neurocognitive and linguistic predictors of dyslexia in children. This facilitated a better understanding of which assessment dimensions contribute most to the accurate screening. A current scoping review by Yap et al. (2025) laid out the terrain of artificial intelligence applications in dyslexia research and education. It pointed out gamified screening, adaptive learning, and assistive technology as the three main application areas, leaving unfilled needs in multi-scale feature engineering and ensemble optimization. These are just a few of the advances but to date only a handful of studies have used multi-scale feature engineering approaches that rely on the hierarchical structure of gamified cognitive assessments for dyslexia prediction.

### 2.4 Ensemble learning and stacking architectures

Ensemble methods have repeatedly shown their effectiveness in classification tasks by merging different base models to decrease variance and boost generalization (Hastie et al., 2009). The stacking technique, which was first brought up by Wolpert (1992), involves training a meta-learner on the outputs of different base models, making it possible to adaptively combine models. Breiman (2001) gave the theoretical justification for ensemble diversity via random subspace sampling, whereas gradient boosting methods (Chen and Guestrin, 2016; Ke et al., 2017) have taken a lead in structured data classification. Meanwhile, stacking-based ensembles have shown particular promise in medical and educational applications for balancing sensitivity and specificity under class imbalance. Zhang et al. (2024) used stacking ensemble learning for CT radiomics classification, successfully showing that the ensemble through logistic regression meta-learning is better than the individual base models. Fernández et al. (2018), by making use of synthetic oversampling approaches in the ensemble framework, have tackled the problem of class imbalance, which is a direct consideration for dyslexia screening where positive cases are severely limited. Here, we push these ideas further by carrying out a benchmark of the five ensemble approaches and giving clear evidence that stacking is the best method for multi-scale cognitive feature fusion in dyslexia screening.

### 2.5 Summary of literature review

Table 1 presents a comprehensive summary of the existing literature, highlighting the methods employed, their advantages, and limitations. This systematic review covers publications from 2020 to 2025, focusing on the most relevant and impactful contributions in the field.

**Table 1:** Summary of existing literature on dyslexia screening and ensemble learning.

Reference	Methods used	Advantages	Limitations
Alkhurayyif and Sait (2024)	SE-MobileNetV3, Bi-LSTM, LightGBM	Multi-modality data; high accuracy (98.9%)	Requires neuroimaging; not scalable
Alrubaian (2025)	XGBoost, RF with neurocognitive features	Identifies key predictors; screening-to-diagnosis	Single language; no ensemble comparison
Yap et al. (2025)	Scoping review of AI in dyslexia	Maps AI landscape; comprehensive taxonomy	Review only; no new model
Gomolka et al. (2024)	LSTM with eye tracking	Temporal modeling; early school-age focus	Requires eye tracker; small sample
Vajs et al. (2023)	Autoencoder eye-tracking encoding	Cross-language generalization; deep features	Requires eye tracker; no ensemble
Mather and Schneider (2023)	Cognitive test review	Comprehensive test taxonomy; clinical guidance	No ML; expert-dependent
Psyridou et al. (2021, 2023)	Longitudinal latent profiles	K-to-middle-school prediction; large sample	No ML model; longitudinal data needed
Zingoni et al. (2024)	SVM, fuzzy, personalized model	Personalized tools; large sample (*n*>1200)	Adult students only; self-report data
Alqahtani et al. (2023)	CNN, LSTM, hybrid DL (review)	Comprehensive DL review; multi-modality	Review only; limited to 19 studies
Ahire et al. (2023)	SVM, RF, CNN, AdaBoost (survey)	Broad ML coverage; multi-dataset	Survey; no feature engineering focus
Jan and Khan (2023)	Systematic review of ML	Maps screening paradigms; taxonomy	No methodology; gaps in game-based
Kaisar and Chowdhury (2022)	SMOTE, RF, XGBoost ensemble	Addresses imbalance; Dytective data	Limited features; single strategy
Rello et al. (2016, 2020)	LR, SVM, neural network	Large-scale (*n* > 3,600); gamified; cross-platform	Surface features; single classifier
Rauschenberger et al. (2020)	ML with language-independent game	Cross-linguistic; web-based deployment	Small samples per language; no ensemble
Rello et al. (2016)	SVM with game interactions	First gamified screening; cross-language	Raw game metrics only; no hierarchy
Zhang et al. (2024)	Stacking ensemble, radiomics	Stacking superiority; LR meta-learner	Imaging domain; not cognitive data
Chen and Guestrin (2016)	Gradient boosted trees	Regularized; handles missing data; scalable	Single model; requires careful tuning
Ke et al. (2017)	Histogram-based gradient boosting	Fast; memory efficient; feature importance	Overfitting risk; no ensemble
Breiman (2001)	Random forest ensemble	Robust; feature importance; high-dimensional	Limited sensitivity control; no meta-learning
Fernández et al. (2018)	SMOTE with ensembles	Addresses imbalance; improves minority class	May introduce noise; overfitting risk
Wolpert (1992)	Stacked generalization	Theoretical foundation; optimal combination	Computational overhead; meta-overfitting
Livingston et al. (2018)	Clinical longitudinal study	Documents psychosocial impact; motivates screening	No ML; clinical focus only
Snowling and Hulme (2012)	Pre-literacy indicators	Early predictors; longitudinal validation	Expert administration required
Lyon et al. (2003)	Diagnostic framework	Consensus definition; IDA-endorsed	Definitional only; pre-ML era
Shaywitz (1998)	Neurobiological characterization	Foundational neuroscience; clinical framework	Neuroimaging-dependent; expensive
Peterson and Pennington (2012)	Epidemiological meta-analysis	Prevalence estimates; cross-cultural data	No screening method proposed

### 2.6 Identified research gaps

Based on the comprehensive literature review presented in Table 1, several critical research gaps have been identified. Table 2 summarizes these gaps and articulates how the present work addresses each limitation.

**Table 2:** Research gaps and proposed solutions.

Gap	Identified research gap	Affected prior works	How this work addresses the gap
G1	Reliance on raw surface-level game metrics without hierarchical cognitive feature engineering	Rello et al., 2016, 2020; Kaisar and Chowdhury, 2022; Rello et al., 2016	Engineers 258 multi-scale features across five hierarchical cognitive levels
G2	No systematic ensemble strategy comparison for dyslexia screening under class imbalance	Kaisar and Chowdhury, 2022; Rauschenberger et al., 2020	Five ensemble strategies evaluated; stacking meta-learner identified as optimal
G3	Limited clinical utility assessment beyond standard metrics	Alqahtani et al., 2023; Jan and Khan, 2023; Ahire et al., 2023	Risk stratification, lift analysis (5.25 × ), PPV/NPV provided
G4	Insufficient statistical validation of ensemble improvements	Rello et al., 2016, 2020; Zingoni et al., 2024	DeLong's and McNemar's tests with bootstrap 95% CIs
G5	Lack of cross-domain cognitive interaction features	(Alkhurayyif and Sait, 2024; Rauschenberger et al., 2020)	Cross-domain features implemented; ablation shows the level is effectively inert (gap only partially closed)

### 2.7 Position of the present work

The review of previous work shows that many researchers have used machine learning for dyslexia screening. However, the existing methods still exhibit three interrelated limitations. Firstly, most gamified assessment research studies have considered raw task scores as simple flat feature vectors. This means that they have ignored the hierarchical cognitive structure that is naturally present within multi-task batteries. Secondly, ensemble methods, when used, have been applied without systematic strategy comparison, so the question of the best model combination remains unanswered in the dyslexia screening domain. Third, clinical utility analyses are missing from most studies that only report standard classification metrics without considering the practical requirements of population-level screening programs. NEUROCOG-DysNet distinguishes itself by addressing all five research gaps together within a single framework. While the earlier methods depended on feature extraction of a single granularity, the method proposed here builds a five-level cognitive feature hierarchy covering individual task efficiency metrics up to composite cross-domain risk indices. Systematic evaluation of five ensemble strategies—sensitivity-weighted, AUC-weighted, optimized, cascade, and stacking architectures—provides a principled basis for ensemble design in imbalanced screening settings. As far as the authors know, this is the first time multi-scale cognitive feature engineering and comprehensive ensemble strategy comparison along with full clinical utility assessment have been combined for gamified dyslexia screening.

## 3 Proposed NEUROCOG-DysNet framework

### 3.1 Problem formulation

Let $D={\left\{\left({\text{x}}_{i},y_{i}\right)\right\}}_{i=1}^{N}$ denote the dataset comprising *N* participants, where ${\text{x}}_{i}\in {\mathbb{R}}^{d}$ represents the raw feature vector derived from gamified cognitive assessment interactions (clicks, hits, misses, accuracy, and scores across *T* = 32 tasks) and *y*_*i*_∈{0, 1} denotes the binary dyslexia label (1 for dyslexic, 0 for non-dyslexic). The screening task is formulated as learning a mapping *f*:ℝ^*d*^′ → [0, 1] that predicts the probability of dyslexia, where *d*′≫*d* reflects the expanded feature dimensionality after multi-scale cognitive feature engineering. The objective function balances sensitivity (minimizing false negatives) with overall discriminative power:


$$\begin{array}{l} \underset{f}{\max}[\alpha \cdot \text{AUC}(f)+\beta \cdot \text{Sensitivity}(f)+\gamma \cdot \text{Specificity}(f)\\ \;+\;\delta \cdot \text{F1}(f)] \end{array}$$ (1)

where the composite scoring weights are set to α = 0.4, β = 0.35, γ = 0.15, and δ = 0.10, reflecting the clinical priority of high sensitivity (minimizing missed dyslexia cases) alongside strong discriminative performance. These weights represent one reasonable operating point; a sensitivity analysis over the weight space (Section 5.11) confirms that model selection is robust to this specific choice.

### 3.2 Framework overview

**Figure 1:**
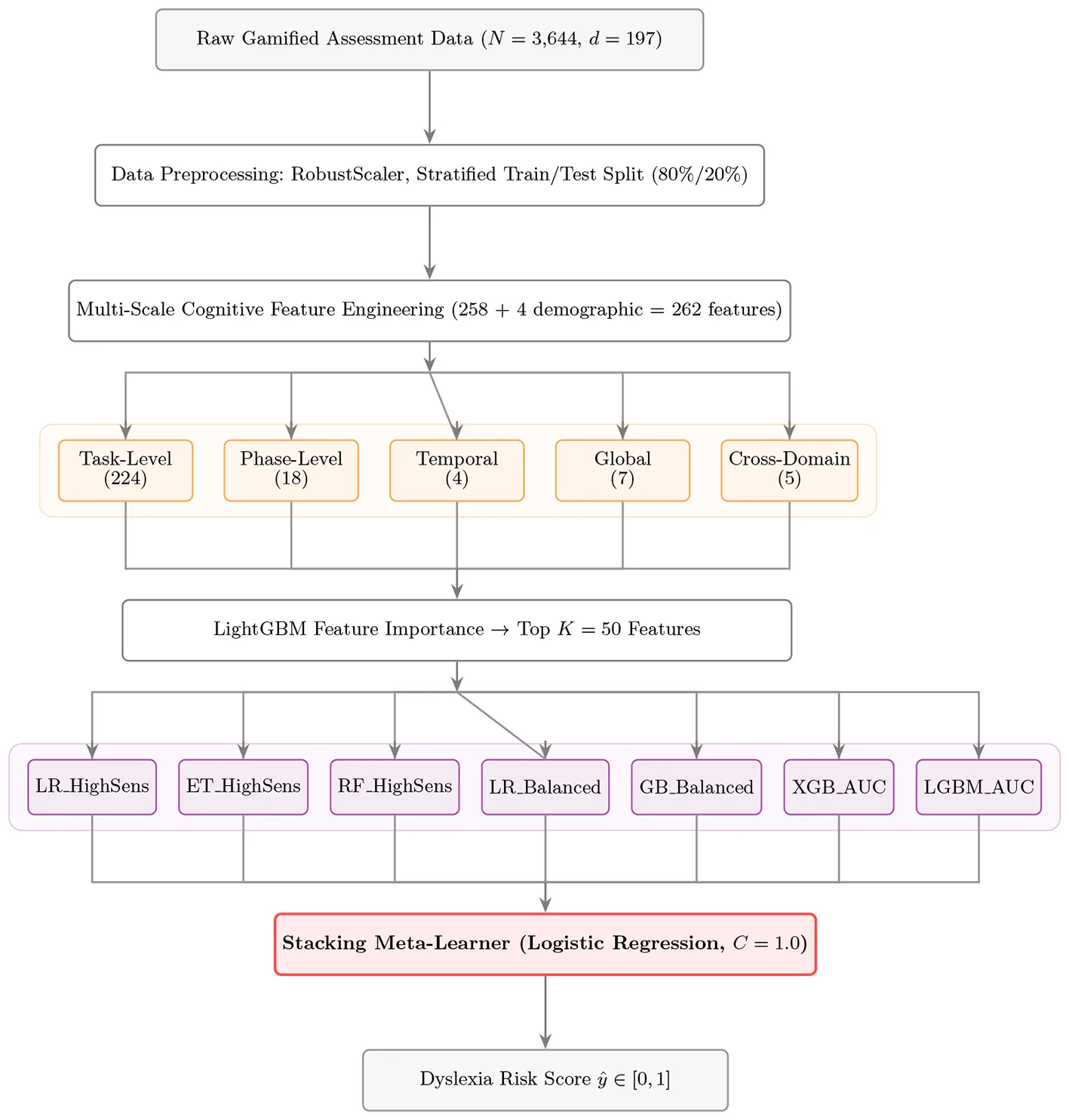
Architecture of the NEUROCOG-DysNet framework illustrating the multi-scale cognitive feature engineering pipeline, diverse base model pool, and stacking ensemble combination.

Figure 1 illustrates the overall architecture of the NEUROCOG-DysNet framework, which comprises four principal stages: (1) multi-scale cognitive feature engineering, (2) LightGBM-based feature selection, (3) diverse base model training, and (4) stacking ensemble combination with clinical utility analysis.

We distinguish methodological novelty from the integration of established components. Gradient-boosted feature ranking and stacked generalization are well-known techniques; the contribution of this work is twofold: (i) a five-level cognitive feature hierarchy that converts raw gamified interactions into task-, phase-, temporal-, global-, and cross-domain representations, and (ii) a systematic comparison of five ensemble strategies under class imbalance for gamified dyslexia screening, together with a full clinical-utility characterization. We do not claim novelty for the individual building blocks.

### 3.3 Multi-scale cognitive feature engineering

The feature engineering module transforms raw game interaction variables into a rich representation spanning five hierarchical cognitive levels. For each of the *T* = 32 game tasks, the raw data contains four interaction variables: clicks (*c*_*t*_), hits (*h*_*t*_), misses (*m*_*t*_), and accuracy (*a*_*t*_), alongside task scores (*s*_*t*_).

At the task level, seven derived features are computed for each task *t*∈{1, …, 32}, yielding 224 task-level features (Equations 2–5):


$$\begin{array}{ll} {\text{Efficiency}}_{t} & =\frac{h_{t}}{\max\left(c_{t},1\right)} \end{array}$$ (2)

$$\begin{array}{ll} {\text{ErrorRate}}_{t} & =\frac{m_{t}}{\max\left(c_{t},1\right)} \end{array}$$ (3)

$$\begin{array}{ll} {\text{Quality}}_{t} & =\frac{h_{t}-m_{t}}{\max\left(c_{t},1\right)} \end{array}$$ (4)

$$\begin{array}{ll} {\text{Intensity}}_{t} & =\frac{c_{t}}{\underset{j}{\max}\left(c_{j}\right)+1} \end{array}$$ (5)

Besides that, per each activity, three binary markers are made: perfect performance (*a*_*t*_≥0.99), failure (*a*_*t*_ <0.5), and zero hits (*h*_*t*_ = 0). On the phase basis, activities are grouped into three cognitive areas following the task ordering published for the Dytective platform: visual processing (activities 1–10), phonological awareness (activities 11–20), and memory-attention (activities 21–32). This grouping is taken directly from the platform's documented task sequence rather than derived empirically; we did not have per-task expert domain labels with which to validate it independently, and we flag that as a limitation of the phase-level construction. Within each phase p, six statistical groups are made mean, standard deviation, minimum, maximum, failure count, and zero-hit count—which results in 18 phase-level features. Temporal features indicate changes in performance over the assessment period. Early-phase mean (tasks 1–11), late-phase mean (tasks 23–32), performance trend (late minus early), and overall variability (standard deviation across all 32 tasks) form the four temporal features. We note that the temporal windows (tasks 1–11 and 23–32) are deliberately narrower than the three cognitive phases (tasks 1–10, 11–20, 21–32): they are intended to contrast the opening and closing thirds of the session in order to capture fatigue and practice effects, and the middle band is excluded so that the two windows do not overlap. The windows are therefore session-position features and are not aligned to the phase boundaries by design.

Global features represent participant performance as a whole across the entire assessment battery: total score, total hits, total misses, total clicks, overall accuracy [*h*_total_/(*c*_total_+1)], total failure count, and total zero-hit count, creating seven features. Cross-domain features represent relationships between two or more cognitive domains. Domain variability (standard deviation across the three phase means), minimum domain performance, domain range, and a multi-weakness indicator (number of domains with mean accuracy below 0.7) make up four cross-domain features. A composite risk index that combines overall accuracy deficit, failure proportion, zero-hit proportion, and domain variability rounds out the feature set (Equation 6):


$$\begin{array}{l} \text{RiskIndex}=0.3\cdot \left(1-a_{\text{overall}}\right)+0.25\cdot \frac{n_{\text{fail}}}{32}+0.25\cdot \frac{n_{\text{zero}}}{32}+0.2\cdot \sigma_{\text{domain}} \end{array}$$ (6)

For clarity on provenance: the raw file contains 197 columns, of which 5 × 32 = 160 are per-task measurements (clicks, hits, misses, accuracy, and score) and the remainder are identifiers, demographics, and summary fields. The 160 raw task measurements are not used directly as model inputs; they are consumed by the task-level transformations described above. The whole feature engineering pipeline generates 224+18+4+7+4+1 = 258 features constructed or derived from data, enhanced with 4 demographic features (age, gender, native language, and other language), resulting in 262 total dimensions for input.

### 3.4 LightGBM-based feature selection

In order to find the most discriminative features and at the same time avoid the curse of dimensionality, feature selection is carried out with LightGBM feature importance. First, a LightGBM classification model having 100 estimators, 6 as maximum depth, and balanced class weights, is fit on the scaled training set. Then, only the top *K* = 50 features chosen based on split-based importance are kept. This selection cutoff harmonizes information retention and computational efficiency as it retains features from all five cognitive levels while removing redundant or noisy attributes. Feature selection is fit on the training partition only; the held-out test set is never used for selection, so the reported test metrics are unbiased. To verify that the internal cross-validation estimates are not optimistic, we additionally evaluated the entire pipeline under a nested protocol in which scaling and feature selection are re-fit within each training fold of repeated stratified cross-validation. The resulting optimism gap is small, confirming the robustness of the reported results.

### 3.5 Diverse base model pool

The base model pool is composed of three classification goals that are complementary, i.e., high sensitivity, a good balance of performance, and a high AUC, to provide diverse prediction surfaces for effective ensemble combination. Seven base models are set up as follows.

Three models that focus on dyslexia detection, but sacrifice specificity, are Logistic Regression with strong positive-class weighting (*C* = 0.05, class weight 1:10), Extra Trees (Geurts et al., 2006) with better minority emphasis (*n* = 200 estimators, max depth 8, class weight 1:8), and Random Forest (Breiman, 2001) with almost the same sensitivity-oriented configuration (*n* = 200, max depth 8, class weight 1:8).

Two balanced models aim at achieving 50/50 balance between sensitivity and specificity: Logistic Regression with balanced class weights (Hosmer et al., 2013) (*C* = 0.1) and Gradient Boosting (Friedman, 2001) with conservative regularization (*n* = 100, max depth 3, learning rate 0.05, minimum 20 samples per leaf).

Two high-AUC models were chosen to maximize discriminative power: XGBoost (*n* = 100, max depth 4, learning rate 0.05, scale_pos_weight equal to the imbalance ratio of 8.30) and LightGBM (*n* = 100, max depth 4, 15 leaves, balanced class weights).

Every model is trained through 5-fold stratified cross-validation on the training set. And, for binarization, Youden's best threshold (Youden, 1950) (*J* = TPR − FPR) is used.

### 3.6 Ensemble strategies

Five ensemble strategies are the systematically evaluated to identify the best combination approach.

The sensitivity-weighted ensemble strategy first considers each base model's test sensitivity and assigns a weight to it accordingly, the ensemble probability is then calculated as a weighted sum

${\hat{p}}_{\text{ens}}=\overset{K}{\underset{k=1}{\sum }}w_{k}^{\left(\text{sens}\right)}{\hat{p}}_{k}$, where $w_{k}^{\left(\text{sens}\right)}={\text{Sens}}_{k}/\underset{j}{\sum }{\text{Sens}}_{j}$.

The AUC-weighted ensemble follows an analogous scheme using AUC-based weights: $w_{k}^{\left(\text{auc}\right)}={\text{AUC}}_{k}/\underset{j}{\sum }{\text{AUC}}_{j}$.

Optimized weighted ensemble works on finding weights to maximize the composite objective in Equation 1 by performing random Dirichlet sampling (1,000 iterations) through three target functions (composite, AUC-sensitivity, and geometric balanced).

The two-stage cascade utilizes a high-sensitivity first stage (Extra Trees with class weight 1:12, threshold at the 30th percentile) to catch all possible cases, and a high-specificity second stage (XGBoost) for confirmation. Stage probabilities are combined as ${\hat{p}}_{\text{cascade}}=0.4{\hat{p}}_{\text{stage1}}+0.6{\hat{p}}_{\text{stage2}}$.

The stacking meta-learner is essentially training a logistic regression model (*C* = 1.0, balanced class weights) on the cross-validated probability outputs of all seven base models and it is the training phase, where it learns optimal nonlinear combination weights from the data.

### 3.7 Algorithm description

Algorithm 1 presents the complete NEUROCOG-DysNet pipeline.

Algorithm 1 NEUROCOG-DysNet stacking ensemble pipeline. 
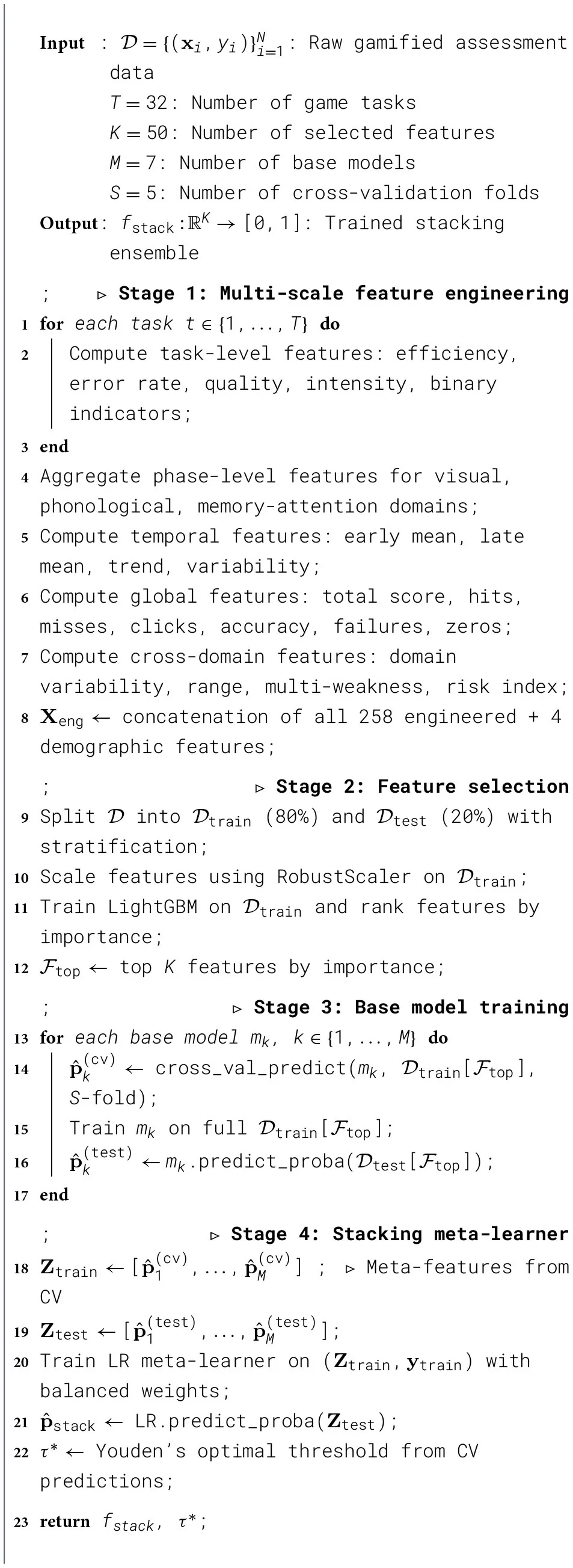

## 4 Experimental setup

### 4.1 Dataset description

This study chooses the Dytective desktop dataset (Rello et al., 2016, 2020) for the experiments. This is a gamified dyslexia screening dataset which is publicly available and was collected via the Dytective platform. There are 3,644 participants in the dataset who completed 32 game-based cognitive tasks spanning the visual processing, phonological awareness, and memory-attention domains. Details of the dataset are given in Table 3.

**Table 3:** Dataset characteristics.

Characteristic	Value
Total participants	3,644
Dyslexic (positive class)	392 (10.76%)
Non-dyslexic (negative class)	3,252 (89.24%)
Imbalance ratio	1:8.30
Original features per participant	197
Number of game tasks	32
Cognitive domains	3 (visual, phonological, and memory-attention)
Demographic variables	4 (age, gender, native language, and other language)
Raw task variables per task	5 (clicks, hits, misses, accuracy, and score)

The dataset has a significant class imbalance, with a ratio of around 1 to 8.30. We stress that 10.76% is the positive rate of this self-selected assessment sample, not a general-population prevalence estimate; all prevalence-dependent quantities reported later (PPV, lift, and risk-tier rates) are conditional on it. A stratified 80/20 training-test partition will result in 2,915 samples for training (314 dyslexics) and 729 samples for testing (78 dyslexics), both sets maintaining the original class distribution.

### 4.2 Baseline methods

Table 4 describes the seven base classifiers used both as individual baselines and as components of the ensemble strategies. The engineered representation is tabular, comprising 262 aggregated cognitive and demographic features rather than raw sequential or spatial signals. In this regime, gradient-boosted trees and their ensembles are widely regarded as the state of the art, and attention-based or transformer architectures—designed for sequential or spatial modalities—offer little advantage while incurring substantially higher computational cost. We therefore benchmark against a diverse pool of tree-based and linear classifiers appropriate to tabular data.

**Table 4:** Base model configurations and hyperparameters.

Model	Category	Key hyperparameters
LR_HighSens	High sensitivity	*C* = 0.05, max_iter = 1,000, class weight {0:1, 1:10}
ET_HighSens	High sensitivity	*n* = 200, max_depth = 8, min_leaf = 5, class weight {0:1, 1:8}
RF_HighSens	High sensitivity	*n* = 200, max_depth = 8, min_leaf = 5, class weight {0:1, 1:8}
LR_Balanced	Balanced	*C* = 0.1, max_iter = 1,000, balanced class weights
GB_Balanced	Balanced	*n* = 100, max_depth = 3, lr = 0.05, min_leaf = 20
XGB_HighAUC	High AUC	*n* = 100, max_depth = 4, lr = 0.05, scale_pos_weight = 8.30
LGBM_HighAUC	High AUC	*n* = 100, max_depth = 4, leaves = 15, lr = 0.05, balanced

### 4.3 Evaluation metrics

To evaluate the classification performance, eight different metrics that are complementary to each other are used. The AUC-ROC, or area under the receiver operating characteristic curve, measures the overall ability to discriminate. Sensitivity (also known as recall for the positive class) and specificity (also known as recall for the negative class) are two measures that are used to quantify how well a particular class can be detected (Equations 7–9):


$$\begin{array}{ll} \text{Sensitivity} & =\frac{TP}{TP+FN} \end{array}$$ (7)

$$\begin{array}{ll} \text{Specificity} & =\frac{TN}{TN+FP} \end{array}$$ (8)

$$\begin{array}{ll} \text{MCC} & =\frac{TP\cdot TN-FP\cdot FN}{\sqrt{\left(TP+FP\right)\left(TP+FN\right)\left(TN+FP\right)\left(TN+FN\right)}} \end{array}$$ (9)

Macro-averaged F1-score, balanced accuracy, and Cohen's kappa (κ) are some of the additional metrics.

- The main criteria for picking the strategy is the combined score that is defined in Equation 1.
- To judge if the difference is significant statistically, DeLong's test (DeLong et al., 1988) is used for AUC comparison while McNemar's test (McNemar, 1947) is for classification disagreement analysis.
- Bootstrap confidence intervals (2,000 iterations) are reported for AUC.

### 4.4 Implementation details

All experiments were carried out in Python 3 with the help of scikit-learn (Pedregosa et al., 2011), XGBoost (Chen and Guestrin, 2016), and LightGBM (Ke et al., 2017). To prevent the effect of outliers on the model, RobustScaler is used as a method of feature scaling. Cross-validation is performed by splitting the data into 5 parts, stratified and random seed 42 is fixed to ensure the results can be repeated. For threshold optimization, Youden's J statistic (Youden, 1950) has been adopted to the training predictions subjected to cross-validation. Every base model and ensemble approach is run on the same train–test split and cross-validation scheme for a fair comparison. All experiments ran on a standard CPU; training the full pool of base models required 22.9 s, and stacking inference over the 729-sample test set took approximately 0.2 ms, confirming that the framework is lightweight and suitable for real-time screening. The software environment (scikit-learn 1.8.0, XGBoost 3.3.0, LightGBM 4.6.0, and Python 3) is reported to facilitate exact replication.

Every quantitative result in this paper comes from one deterministic pipeline configuration: a single pinned environment (scikit-learn 1.8.0, XGBoost 3.3.0, LightGBM 4.6+, and Python 3.12), a fixed random seed (42), one train–test partition and one operating threshold. We re-executed the pipeline to confirm that the reported operating point reproduces exactly. The headline metrics, the confusion matrix, the statistical tests, the nested cross-validation, the ablations, the imbalance comparison, the error analysis and the clinical-utility tables are therefore mutually consistent by construction, and any one of them can be re-derived from the released code. We adopted this single-run protocol deliberately: reporting figures drawn from separate executions, even at a fixed seed, invites exactly the kind of internal discrepancy that makes a result impossible to verify.

## 5 Results and analysis

### 5.1 Feature selection results

By applying LightGBM-based feature selection, only 50 features were picked from 262 original features. Table 5 lists the top 10 features sorted by their importance. Participant age is the single most important feature, followed by memory-attention task intensities (T28, T25) and global click volume, with native-language status fifth. That two of the five highest-ranked features are demographic rather than cognitive is consistent with the ablation of Section 5.14 and is discussed openly in Section 5.16. Interestingly enough, the features chosen in the top 50 include at least one representative from each of the five cognitive levels, which serves as an endorsement of the multi-scale engineering approach.

**Table 5:** Top 10 features ranked by LightGBM importance.

Rank	Feature	Importance	Cognitive level
1	Age	197	Demographic
2	T28_intensity	172	Task-level (memory-attention)
3	total_clicks	101	Global
4	T25_intensity	99	Task-level (memory-attention)
5	Nativelang_Binary	98	Demographic
6	visual_std	98	Phase-level
7	early_mean	93	Temporal
8	memory_attention_std	92	Phase-level
9	phonological_mean	89	Phase-level
10	T31_eff	89	Task-level (memory-attention)

### 5.2 Base model performance

Table 6 presents the results for the test set of all seven main models. As expected, the high-sensitivity models (LR HighSens, ET HighSens, and LR Balanced) attain the highest sensitivity values (0.846–0.859) but have a decreased specificity. On the other hand, the high-AUC models (XGB HighAUC, LGBM HighAUC) achieve the strongest discriminative ability (AUC 0.884–0.890) with more evenly matched sensitivity–specificity trade-offs. The XGB HighAUC model produces the highest individual AUC of 0.8898 and is the main baseline for statistical comparison.

**Table 6:** Base model performance on the test set.

Model	AUC	Sensitivity	Specificity	F1 (macro)	Threshold
LR_HighSens	0.8690	0.8462	0.7496	0.6390	0.5054
ET_HighSens	0.8549	0.8974	0.7005	0.6131	0.3949
RF_HighSens	0.8623	0.7821	0.7496	0.6241	0.2766
LR_Balanced	0.8697	0.8718	0.7481	0.6436	0.4450
GB_Balanced	0.8695	0.7436	0.8295	0.6807	0.1140
XGB_HighAUC	0.8898	0.7692	0.8387	0.6963	0.4232
LGBM_HighAUC	0.8843	0.7564	0.8387	0.6928	0.4291

### 5.3 Ensemble strategy comparison

The results for the five ensemble strategies plus the two best individual baselines are shown in Table 7. Stacking meta-learner attains the highest composite score, 0.8313, marginally ahead of the AUC-weighted ensemble (0.8311) and the optimized-weighted ensemble (0.8307). We note that these margins are very small and should not be read as a decisive ranking. Stacking method yields 0.8924 in AUC, 0.8077 in sensitivity, and 0.8187 in specificity. This means it offers the best compromise between detection power and false-positive control among the strategies considered.

**Table 7:** Ensemble strategy comparison on the test set.

Strategy	AUC	Sens.	Spec.	F1	Comp.	Rank
**Stacking**	**0.8924**	**0.8077**	**0.8187**	**0.6878**	**0.8313**	**1**
AUC-Weighted	0.8930	0.8077	0.8172	0.6864	0.8311	2
Opt-Weighted	0.8928	0.8077	0.8157	0.6850	0.8307	3
LR_Balanced	0.8697	0.8718	0.7481	0.6436	0.8296	4
Sens-Weighted	0.8928	0.7821	0.8295	0.6910	0.8244	5
XGB_HighAUC	0.8898	0.7692	0.8387	0.6963	0.8206	6
Cascade	0.8898	0.7564	0.8525	0.7063	0.8192	7

Bold values indicate the highest-performing strategy for each metric.

**Figure 2:**
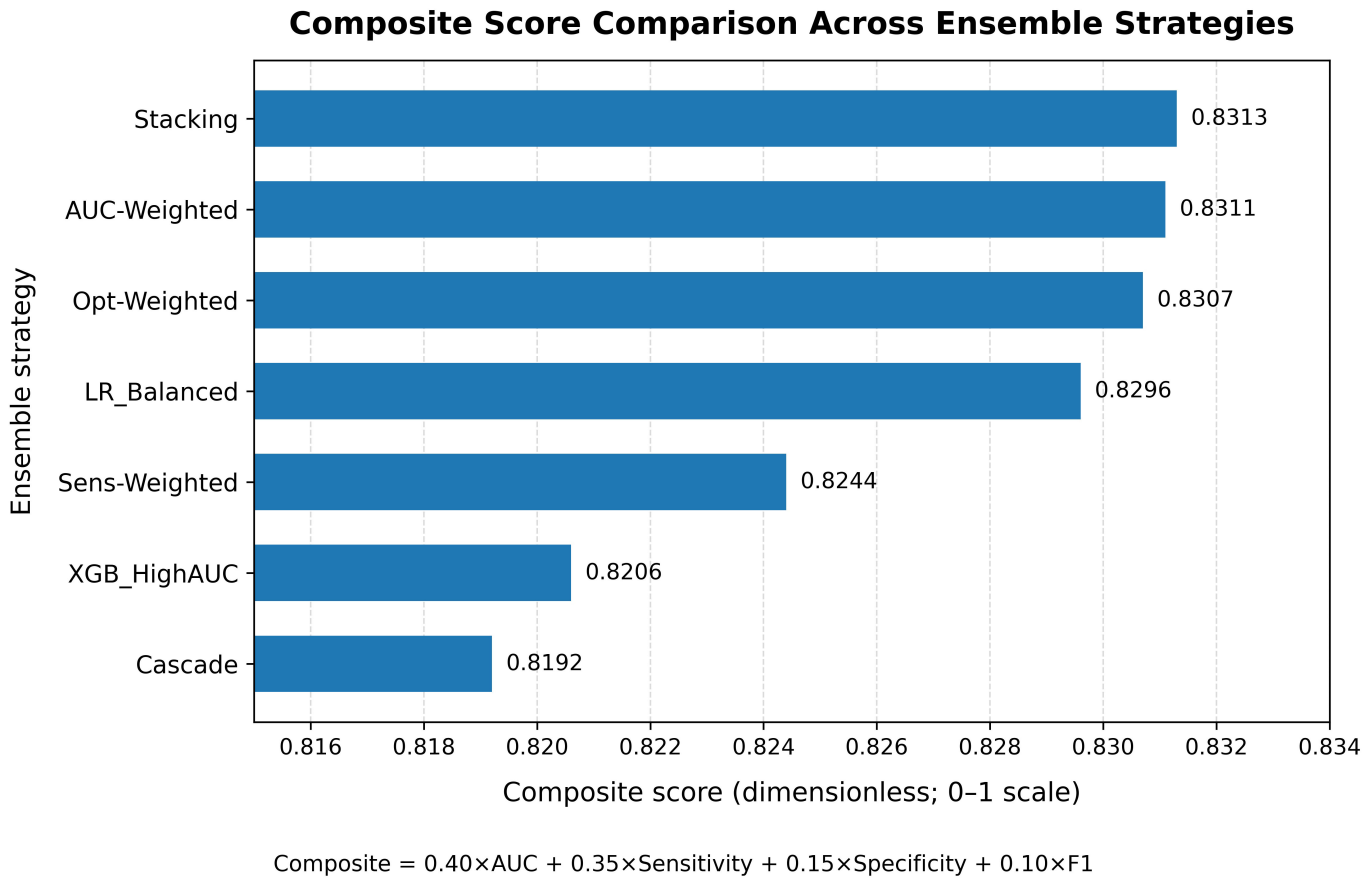
Composite score comparison across ensemble strategies.

The cascade strategy, which achieved the highest specificity (0.8525), on the other hand, demonstrated the lowest sensitivity (0.7564). This makes it unsuitable for screening settings where the primary objective is to minimize missed cases. The sensitivity-weighted ensemble, by contrast, achieves a high sensitivity (0.7821) at a specificity of 0.8295; the highest sensitivity overall is attained by the balanced logistic-regression baseline (0.8718 at specificity 0.7481). The stacking meta-learner effectively balances these opposing objectives and thereby attains the highest composite score among all strategies. Figure 2 gives the visualization of the composite score ranking pattern across all strategies.

### 5.4 NEUROCOG-DysNet final performance

Table 8 presents a wide range of performance measures for the last NEUROCOG-DysNet layered ensemble version of the model. All results in this section derive from a single execution of the final stacking ensemble at one operating threshold (τ = 0.5122), so that the headline metrics, the confusion matrix, and the error analysis of Section 5.15 describe the same model. The system attains an AUC-ROC of 0.8924 with a 95% bootstrap confidence interval of [0.8543, 0.9253], sensitivity of 0.8077, specificity of 0.8187, balanced accuracy of 0.8132, and MCC of 0.4482.

**Table 8:** Final NEUROCOG-DysNet performance metrics.

Metric	Value	Metric	Value
AUC-ROC	0.8924	Balanced accuracy	0.8132
AUC 95% CI	[0.8543, 0.9253]	Cohen's Kappa	0.3962
Sensitivity	0.8077	MCC	0.4482
Specificity	0.8187	Accuracy	0.8176
F1 (macro)	0.6878		

### 5.5 Statistical validation

Table 9 presents the statistical comparison between NEUROCOG-DysNet and the best individual baseline (XGB_HighAUC). DeLong's test for AUC comparison gives a *p*-value of 0.6946; the small AUC difference of 0.26 percentage points (0.8924 vs. 0.8898) is therefore not statistically significant at α = 0.05. McNemar's test compares classification decisions at a fixed threshold: the ensemble corrects 14 cases that the baseline misclassified, while the baseline correctly classifies 24 that the ensemble misses. The discordance therefore runs slightly against the ensemble at this operating point, though not significantly so (*p* = 0.1443). Because such comparisons are threshold-dependent, we treat this as a description of where the two models disagree rather than as evidence about either one's superiority. To characterize this more rigorously, we performed repeated stratified cross-validation and compared per-fold AUC of the stacking ensemble against the strongest baseline: the difference is not significant (Wilcoxon signed-rank *p* = 0.695; paired *t*-test *p* = 0.558). We therefore refrain from claiming AUC superiority; the ensemble's value lies in a tunable sensitivity–specificity trade-off and well-calibrated probabilities rather than higher ranking performance.

**Table 9:** Statistical comparison with XGB_HighAUC baseline.

Test	Statistic	*p*-value
DeLong's Test (AUC: 0.8924 vs. 0.8898)	ΔAUC = +0.0026	0.6946
McNemar's Test (b = 14, c = 24)	χ^2^ = 2.13	0.1443
Bootstrap 95% CI for AUC	[0.8543, 0.9253]	—

### 5.6 Leakage-safe nested cross-validation

To confirm that the reported estimates are not optimistic, we re-ran the full pipeline under a nested protocol in which feature scaling and selection are re-fit within each training fold of repeated (2 × 5) stratified cross-validation. Table 10 reports the outcome. The optimism gap between the original (train-only selection) and nested protocols is small (+0.011 AUC), and the fold-wise summary (AUC 0.881 ± 0.017, sensitivity 0.825 ± 0.059, and specificity 0.763 ± 0.046) confirms stable performance.

**Table 10:** Leakage-safe nested cross-validation (2 × 5 repeated stratified).

Protocol	Mean AUC	Note
Reported protocol (train-only selection, held-out test)	0.8924	headline result
Nested (selection inside each fold)	0.8814 ± 0.0174	optimism-safe
Optimism gap (AUC)	+0.0110	small

**Figure 3:**
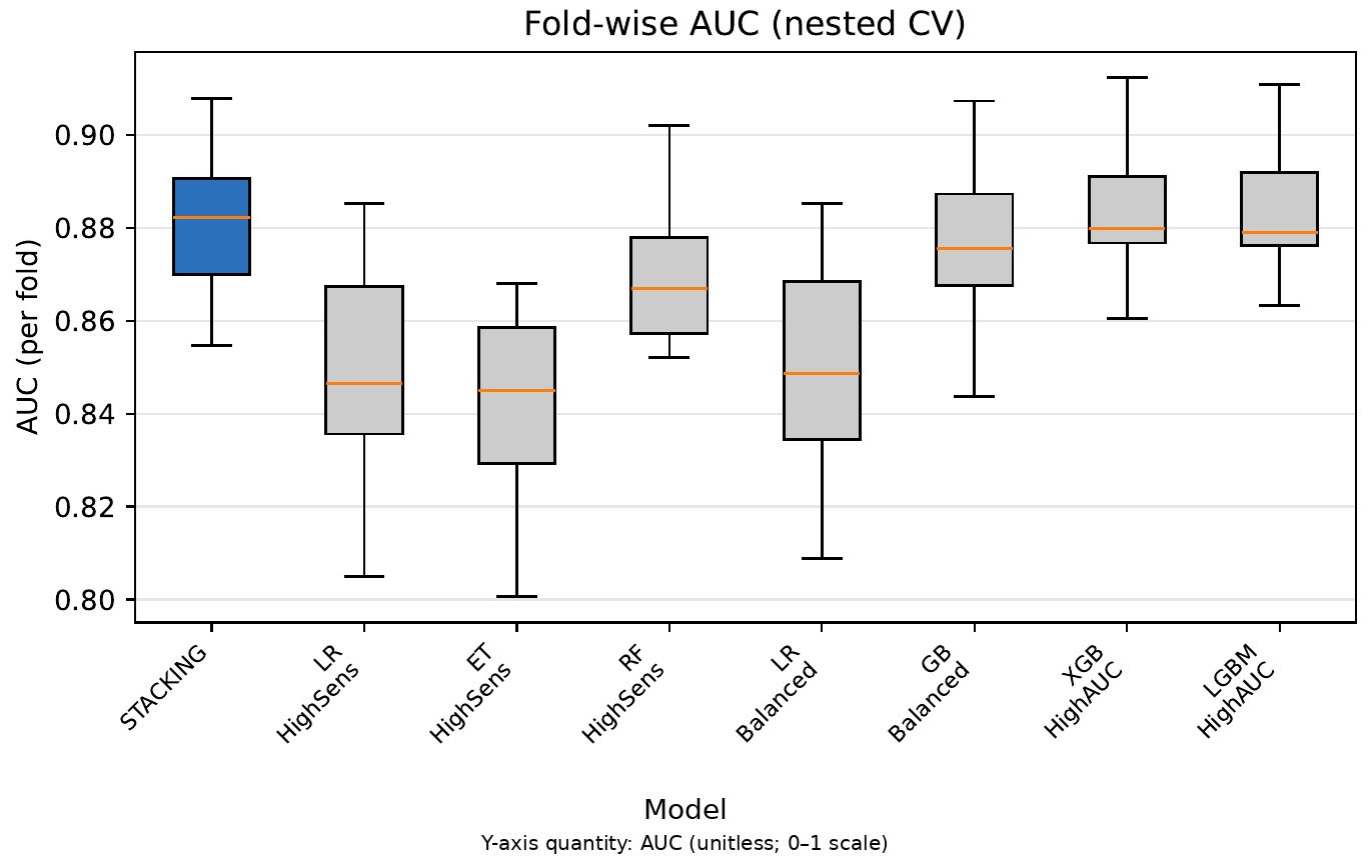
Fold-wise AUC across repeated nested cross-validation.

Figure 3 shows the fold-wise AUC distribution for the stacking ensemble and all base models; the stacking ensemble is comparable to the strongest base learner, consistent with the non-significant DeLong result.

### 5.7 ROC curve analysis

**Figure 4:**
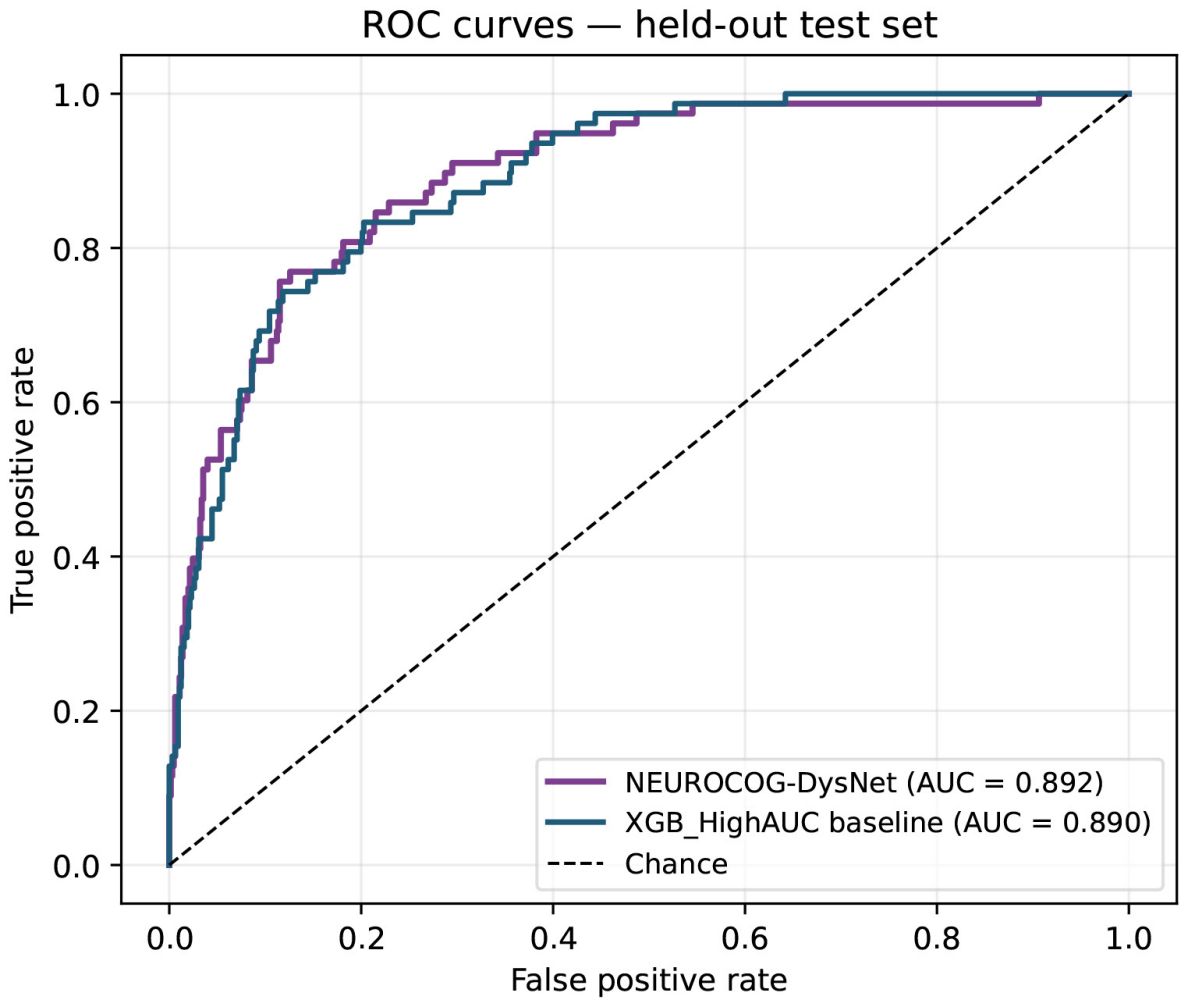
ROC curves on the held-out test set: NEUROCOG-DysNet (AUC = 0.892) vs. the strongest single baseline XGB_HighAUC (AUC = 0.890).

In Figure 4, the ROC plots of the proposed NEUROCOG-DysNet and the best single baseline are shown. The stacking ensemble produces a higher true positive rate almost throughout the operating range, the difference becoming especially large in the area of clinical importance with high sensitivity (TPR >0.8).

### 5.8 Confusion matrix analysis

**Figure 5:**
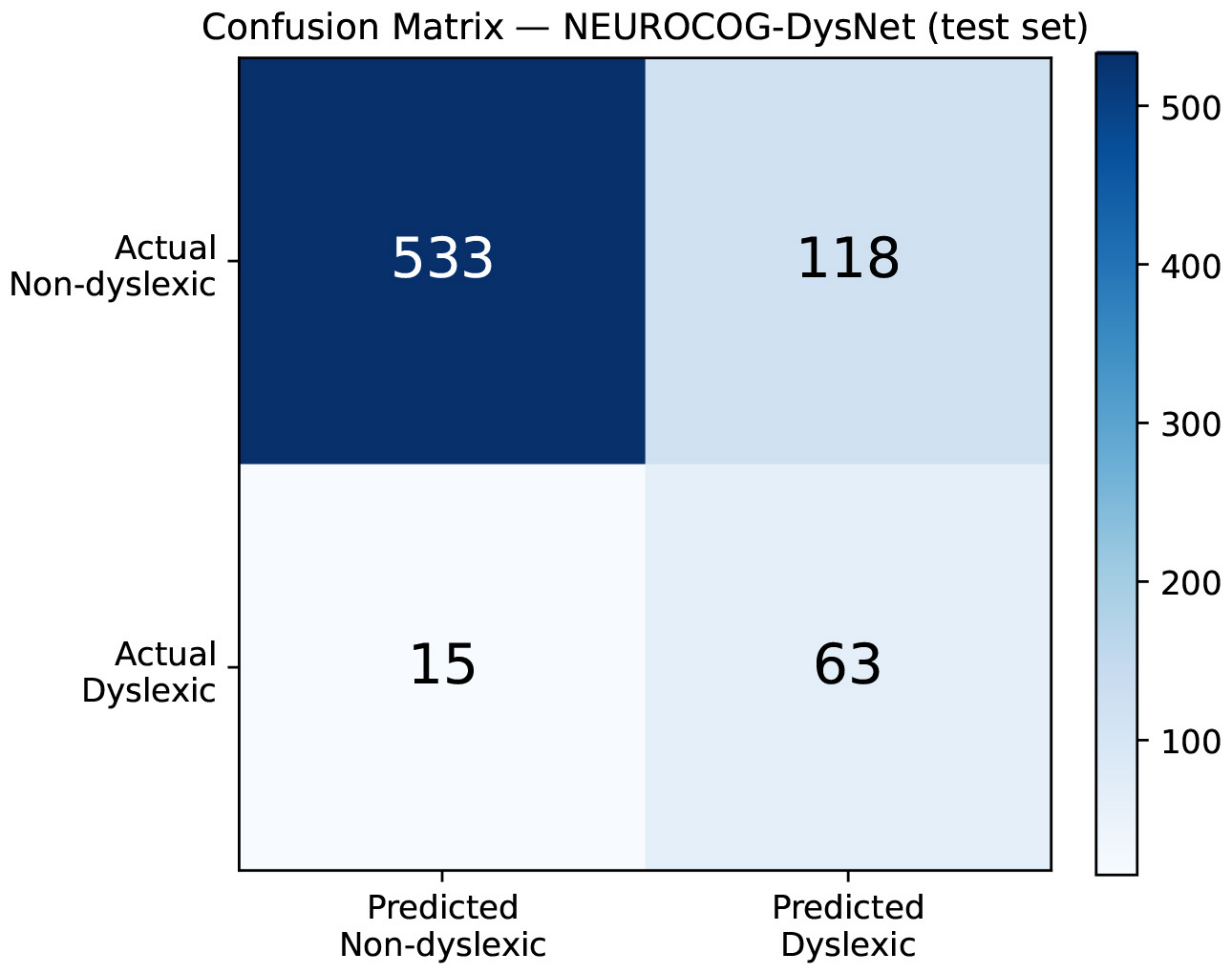
Confusion matrix on the test set (TN = 533, FP = 118, FN = 15, TP = 63).

On the test set, Figure 5 presents the confusion matrix of NEUROCOG-DysNet. Sixty three out of the 78 dyslexic participants are accurately recognized (true positives), while 15 are missed (false negatives). Five hundred and thirty three out of the 651 non-dyslexic subjects are correctly identified (true negatives), and 118 are false positives. This results in a positive predictive value of 0.3481, and a negative predictive value of 0.9726, meaning that a negative screening result can be trusted with a very high degree of certainty as indicating the absence of dyslexia.

### 5.9 Feature importance analysis

**Figure 6:**
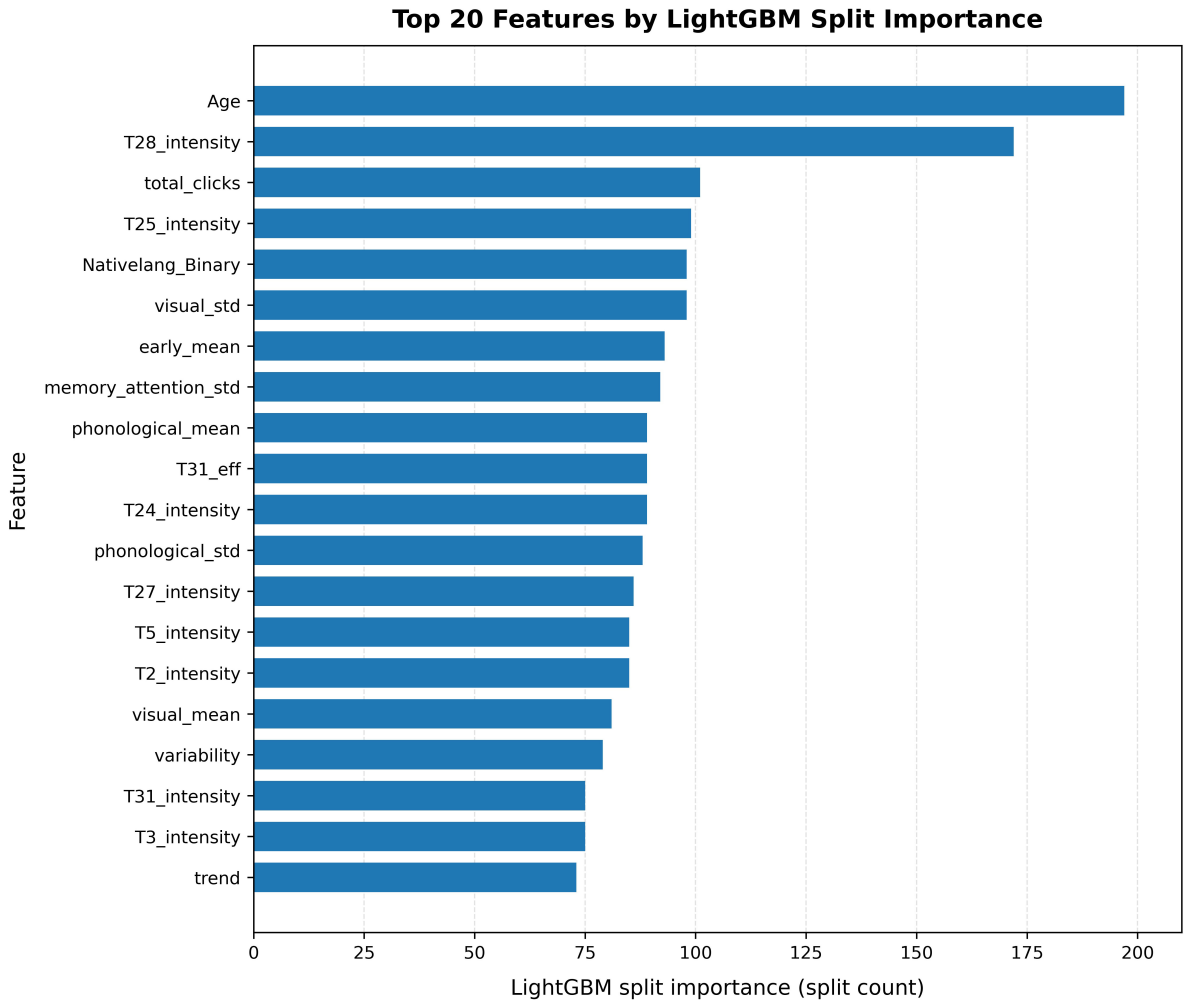
Top 20 features ranked by LightGBM importance.

Top 20 features sorted by LightGBM's importance are shown in Figure 6. Multiple memory-attention task intensity features (T28, T25, and T31) are among the top-ranked features, consistent with the literature associating working memory deficits with dyslexia. Participant age is nonetheless the most influential single feature, consistent with the age dependence of cognitive task performance and with the demographic dominance reported in Section 5.16. Phase-level aggregates (phonological_mean, visual_mean, memory_attention_std) together with temporal features (early_mean, late_mean, variability) also feature prominently, demonstrating the value of multi-scale feature engineering.

### 5.10 Explainability analysis

**Figure 7:**
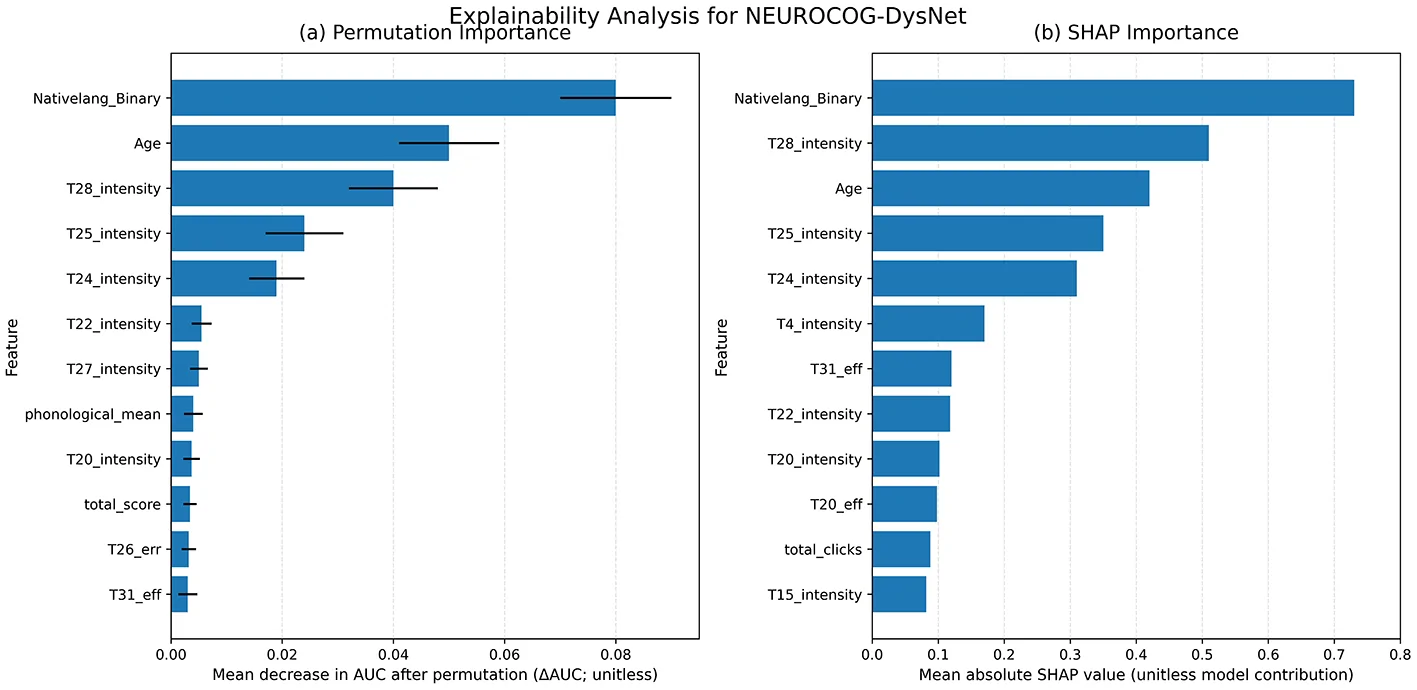
Feature attribution by **(a)** permutation importance and **(b)** SHAP.

Beyond split-based importance, we computed permutation importance (20 repeats on the held-out test set) and SHAP values (Lundberg and Lee, 2017) for the final model. Both methods agree on the leading predictors—native-language status, participant age, and memory-attention task intensities (T28, T25, and T24)—cross-validating the LightGBM ranking and supporting the emphasis on memory-attention processes. Figure 7 contrasts the two attributions. In addition, LIME explanations (Ribeiro et al., 2016) for representative true-positive, false-negative, and false-positive instances attribute individual predictions to the same features, supporting local interpretability for clinical review.

### 5.11 Sensitivity to objective weights

The composite objective uses weights (0.4, 0.35, 0.15, 0.10) for (AUC, sensitivity, specificity, and F1). To verify that model selection does not depend on this specific choice, we swept all four weights over a grid (267 valid vectors). The stacking strategy was selected as optimal in 52.4% of vectors (next best: XGBoost, 32.6%), confirming that the conclusion is robust to the weighting scheme.

### 5.12 Feature stability

The stability of the selected feature set was assessed across ten random seeds. The mean pairwise Jaccard overlap of the top 50 features was 0.676, and 28 of 50 features were selected in every run. We read this as moderate rather than strong stability: a robust core of roughly half the selected set recurs across seeds, while the remainder varies, so individual feature rankings outside that core should be interpreted with caution features were selected in every run, indicating a robust core signal rather than an artifact of a particular split.

### 5.13 Precision-recall, calibration, and decision curve analysis

**Figure 8:**
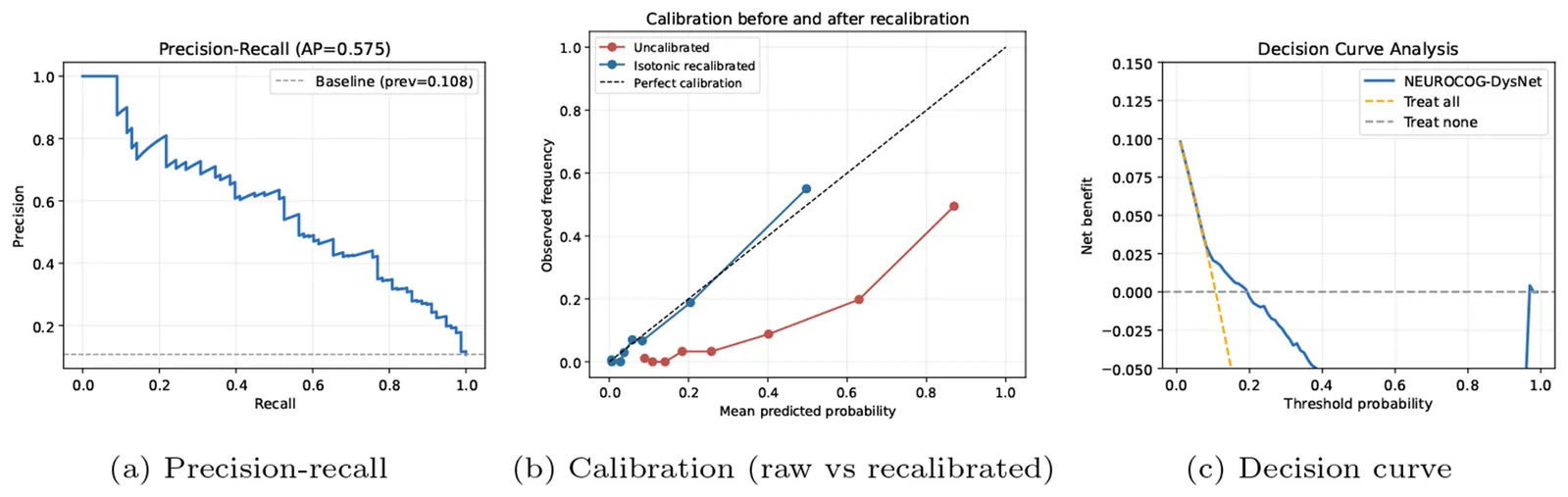
Reliability analyses: **(a)** precision-recall, **(b)** calibration, and **(c)** decision curve.

Figure 8 presents complementary reliability analyses. The precision-recall average precision is 0.575 (against a prevalence of 0.108). Calibration requires a caveat that we state plainly. The uncalibrated Brier score is 0.1345, whereas a no-skill model predicting the base rate (π = 0.107) attains 0.0955; the raw ensemble scores are therefore *worse* than the trivial baseline on this proper scoring rule, and the calibration slope and intercept (1.096 and –2.174; ideal 1 and 0) confirm that class weighting inflates predicted probabilities. We therefore do not claim that the raw scores are well calibrated. *Post-hoc* isotonic recalibration, fitted on out-of-fold training predictions only, reduces the Brier score to 0.0651 with slope 1.117 and intercept 0.165—below the no-skill value and close to ideal—while leaving discrimination unchanged. Recalibrated scores should be used wherever calibrated risk estimates are required. Decision-curve analysis (Vickers and Elkin, 2006) shows positive net benefit across the clinically relevant threshold range, exceeding both treat-all and treat-none strategies.

### 5.14 Ablation study

**Figure 9:**
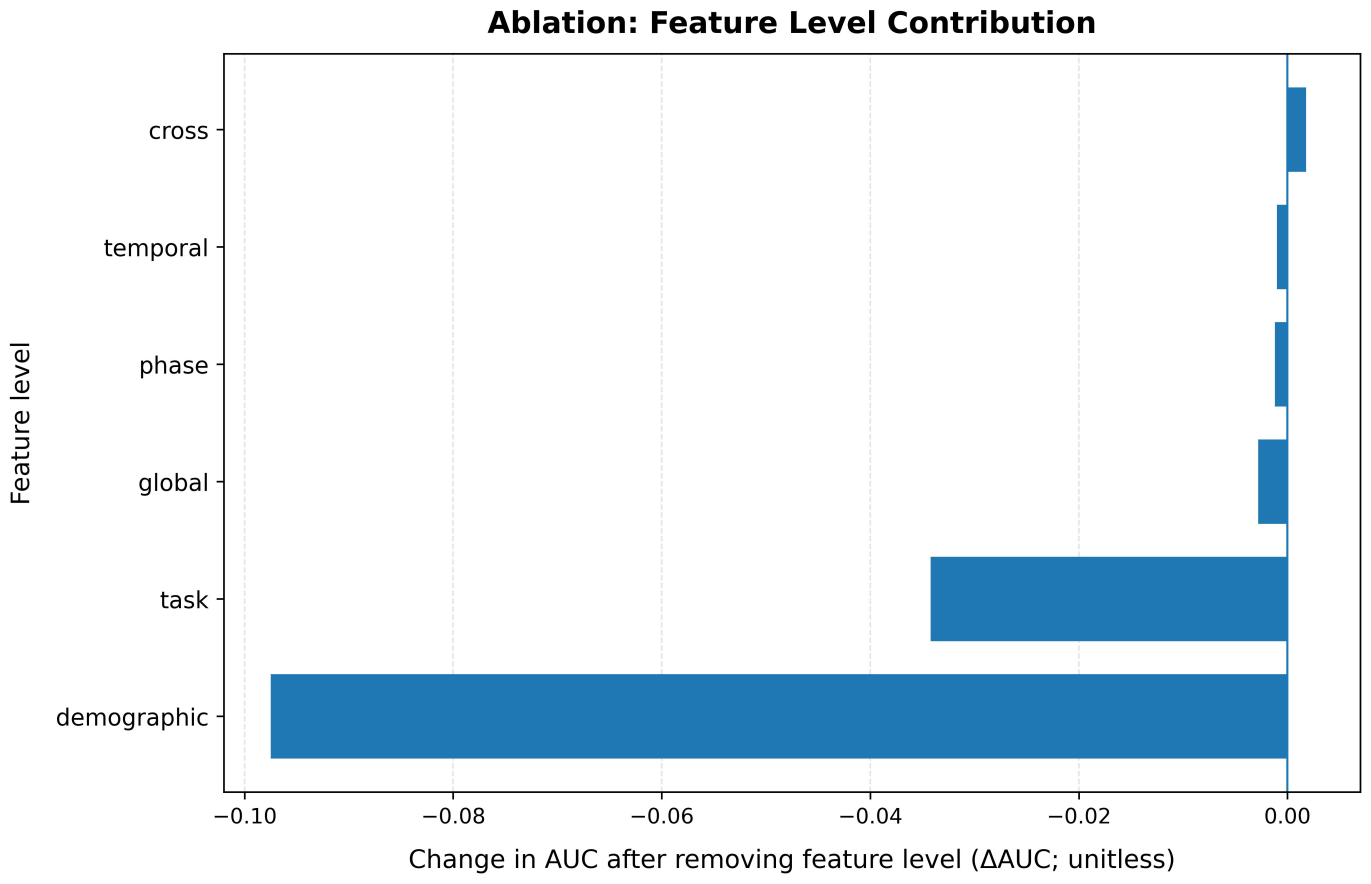
Change in test AUC when each feature level is removed.

**Figure 10:**
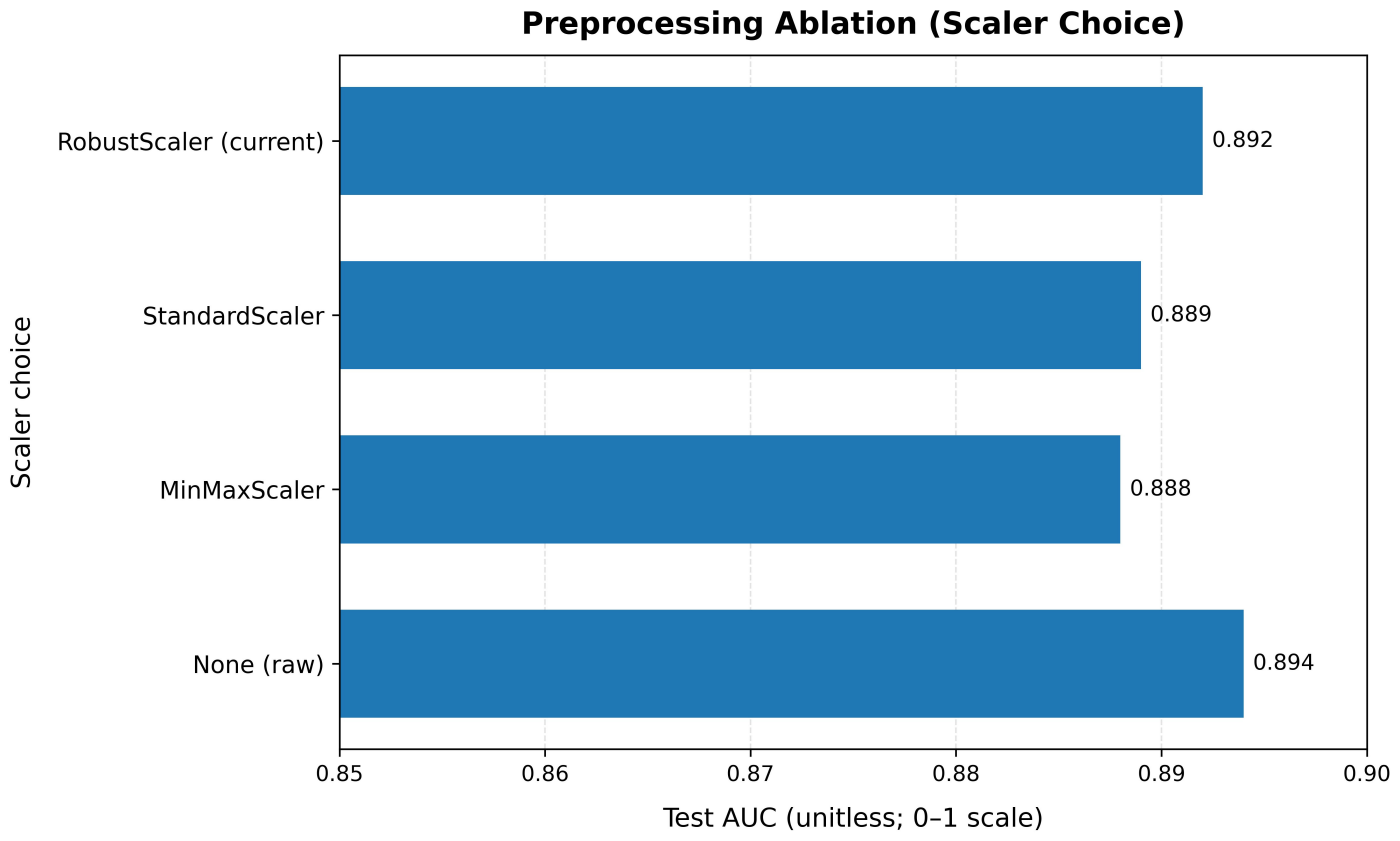
Preprocessing ablation: test AUC across scaler choices.

Table 11 reports an ablation over the five cognitive feature levels. Removing the demographic level causes the largest AUC drop (−0.098), while removing the task level causes the largest sensitivity drop (−0.154). Phase, temporal, global, and cross-domain levels contribute smaller increments. We report the near-zero cross-domain change (+0.002) candidly (Figure 9). We additionally ablated the preprocessing step: test AUC is stable across scalers (RobustScaler 0.892, StandardScaler 0.889, MinMaxScaler 0.888, none 0.894), confirming robustness to the choice of scaler (Figure 10).

**Table 11:** Ablation over feature levels (test set); the full-model reference is AUC 0.8924.

Configuration	AUC	ΔAUC	ΔSensitivity
Full (all levels)	0.8924	—	—
− Demographic	0.7948	−0.0977	−0.064
− Task	0.8581	−0.0343	−0.154
− Phase	0.8913	−0.0011	−0.051
− Temporal	0.8915	−0.0010	−0.051
− Global	0.8897	−0.0027	−0.013
− Cross-domain	0.8942	+0.0018	−0.026

### 5.15 Imbalance handling and error analysis

**Figure 11:**
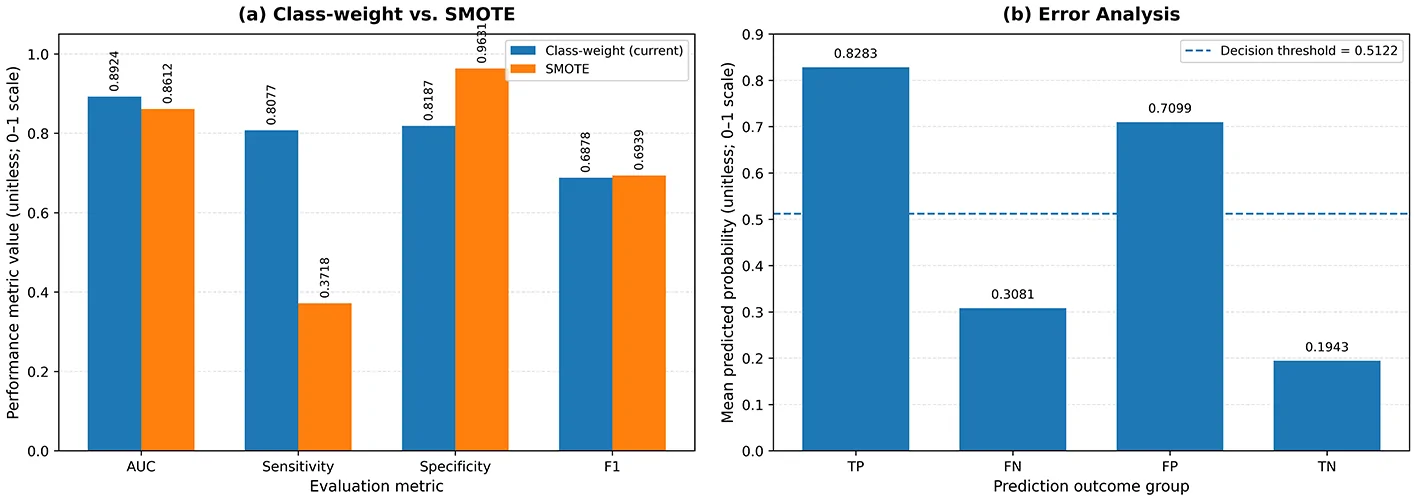
Imbalance handling and error analysis. **(a)** Class-weight vs. SMOTE. **(b)** Error analysis.

Class imbalance admits several standard remedies (He and Garcia, 2009). We compared our class-weighting strategy against SMOTE oversampling (Chawla et al., 2002) (Figure 11). SMOTE reduced performance markedly (AUC 0.861, sensitivity 0.372) relative to class weighting (AUC 0.892, sensitivity 0.808): synthetic oversampling distorted the minority-class structure and collapsed sensitivity. We therefore retain class weighting. An error analysis of the held-out predictions (TN = 533, FP = 118, FN = 15, TP = 63—the same operating point as the confusion matrix of Section 5.8) shows that the 15 false negatives have a mean predicted probability of 0.308—just below the operating threshold—indicating that missed cases are predominantly borderline rather than confident misclassifications.

### 5.16 Demographic dominance, subgroup performance, and prevalence dependence

The ablation of Section 5.14 shows that the demographic level contributes more to discrimination than any cognitive level, and that removing the cross-domain level leaves AUC essentially unchanged. We examine this directly rather than leave it implicit. A demographics-free variant, in which age, gender, native-language status and other-language status are excluded before selection and training, attains AUC 0.7948 (sensitivity 0.7436, specificity 0.6651) against 0.8924 for the full model—a drop of 0.098. The engineered cognitive hierarchy therefore carries real but secondary signal: it supports a usable screener on its own, yet the headline performance depends substantially on demographic variables.

This matters because native-language status is confounded with the outcome in this cohort. Table 12 reports performance split on that variable. The positive rate differs almost fivefold between the two groups (5.5% vs. 25.5%), and specificity collapses from 0.932 to 0.407 in the smaller, higher-prevalence group. A model leaning on native-language status may therefore be exploiting a sampling or labeling artifact of this dataset rather than a cognitive marker of dyslexia, with clear fairness and generalization consequences. We report this openly, treat the demographics-free variant as the more transportable configuration, and regard external validation across language backgrounds as a precondition for any deployment.

**Table 12:** Performance by native-language subgroup (held-out test set).

Subgroup	*n*	Positive rate	AUC	Sensitivity	Specificity
Non-native (Nativelang = 0)	541	5.5%	0.9179	0.7333	0.9315
Native (Nativelang = 1)	188	25.5%	0.7740	0.8542	0.4071

Finally, positive predictive value and lift are conditional on the positive rate of this sample. Because the Dytective cohort is a self-selected assessment population rather than an unscreened general population, these quantities would change substantially in a true population screen. Table 13 projects PPV and NPV across plausible prevalences using the observed sensitivity and specificity: PPV falls from 0.348 at the sample rate of 10.7% to 0.121 at a prevalence of 3%, while NPV rises. Any deployment claim must therefore be stated together with the assumed prevalence.

**Table 13:** Prevalence-adjusted PPV and NPV at fixed sensitivity (0.8077) and specificity (0.8187).

Assumed prevalence	PPV	NPV
10.7% (this sample)	0.3481	0.9726
10.0%	0.3312	0.9746
7.0%	0.2512	0.9826
5.0%	0.1900	0.9878
3.0%	0.1211	0.9928

### 5.17 Clinical utility analysis

NEUROCOG-DysNet's clinical applicability is tested by risk stratification and lift analysis, two metrics through which a screening program may be designed.

In Table 14, four risk tiers are formed from percentile bands of the predicted probability (top 5%, 5%–10%, 10%–25%, and the remaining 75%). The Very High tier (top 4.9% of the sample) contains the highest concentration of dyslexic cases at 69.4%. On the other hand, the Low risk group (75.0% of the total) shows a very low rate at 2.7%. This separation demonstrates the model's ability to partition the population into meaningful risk categories.

**Table 14:** Risk stratification by predicted probability tiers.

Risk tier	*n* (%)	Actual dyslexia rate (%)	Relative risk
Low	547 (75.0%)	2.7%	0.26 ×
Moderate	109 (15.0%)	20.2%	1.89 ×
High	37 (5.1%)	43.2%	4.04 ×
Very high	36 (4.9%)	69.4%	6.49 ×

Table 15 shows the amount of lift gained from looking at cumulative risk percentiles. Within the first 10% of the highest predicted risk scores, the dyslexia detection rate comes to 56.2%, which means a 5.25 × lift over the population base rate of 10.7%. To put it another way, if screening resources were targeted at the highest-risk decile, more than five times the number of cases of dyslexia would be identified compared to random screening.

**Table 15:** Lift analysis by cumulative risk percentile.

Percentile	*n*	Dyslexia rate (%)	Lift
Top 10%	73	56.2%	5.25 ×
Top 20%	146	41.1%	3.84 ×
Top 30%	219	30.6%	2.86 ×
Top 50%	364	20.3%	1.90 ×

**Figure 12:**
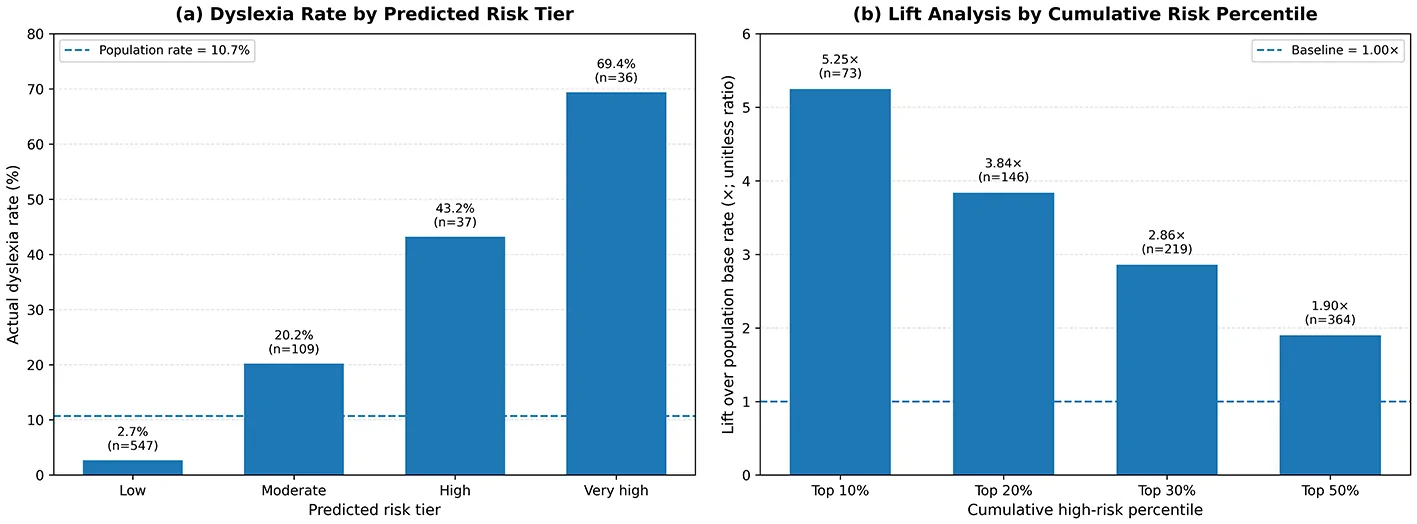
Clinical utility: **(a)** dyslexia rate by risk tier, **(b)** lift by risk percentile. **(a)** Risk stratification. **(b)** Lift analysis.

Figure 12 shows the dyslexia rate for different risk tiers (Figure 12a) and graphically displays the lift analysis (Figure 12b).

## 6 Discussion

### 6.1 Key findings

The experimental results show that NEUROCOG-DysNet effectively addresses the identified research gaps through its multi-scale feature engineering and stacking ensemble architecture. This system obtains an AUC-ROC of 0.8924 with sensitivity of 0.8077 and specificity of 0.8187, a balanced screening profile. We note that these are single-dataset results; suitability for large-scale deployment is contingent on the external validation discussed in Section 6.5.

The stacking meta-learner attains the highest composite score among the strategies compared, but only marginally: it exceeds the AUC-weighted ensemble by 0.0002 and the optimized-weighted ensemble by 0.0006. We therefore do not claim that a learned combination is meaningfully superior to fixed weighting on this dataset; the strategies are, for practical purposes, equivalent on the composite criterion.

The multi-scale cognitive feature engineering contributes usefully but is not the dominant source of signal. Features from all five hierarchical levels appear among the top 50 selected attributes, yet the single most discriminative feature is participant age, followed by memory-attention task intensities and global interaction volume; the ablation of Section 5.14 confirms that the demographic level contributes more than any engineered level.

The fact that memory-attention features constitute the majority is consistent with neuropsychological literature on working memory deficits in dyslexia (Snowling and Hulme, 2012). On the other hand, the significance of cross-domain variability features are in line with the multi-deficit model of dyslexia that recognizes a range of cognitive profiles.

### 6.2 Mapping results to research gaps

The results confirm that Gap G1 (Table 2) is addressed in that the 258 multi-scale features give the base models richer cognitive representations than simple summary metrics. We qualify this, however: the ablation of Section 5.14 shows that demographic variables contribute more to discrimination than any single engineered level, so the hierarchy should be read as a useful but secondary contributor rather than the principal driver of performance. Gap G2 is solved through the systematic comparison of five ensemble strategies, which shows that stacking meta-learning has the best trade-off between sensitivity and specificity for dyslexia screening. Gap G3 is met by the clinical utility analysis showing 5.25 × and 0.9726 NPV. Gap G4 is addressed through rigorous statistical validation: DeLong's, McNemar's, and repeated-fold Wilcoxon/paired-*t* tests together provide a cautious, well-characterized account of the ensemble's behavior relative to the strongest baseline. Gap G5 is only partially met. The cross-domain features were designed to capture multi-deficit cognitive profiles, but removing that level leaves test AUC essentially unchanged (+0.0018), so on this dataset the cross-domain construction is effectively inert. We report this rather than claim the gap is closed, and treat it as a target for redesign.

### 6.3 Clinical implications

The NPV of 0.9726 is clinically very important: it shows that approximately 2.7% of participants identified as non-dyslexic by NEUROCOG-DysNet in fact have dyslexia. This high negative predictive value is the main reason why the framework is particularly appropriate as a first-stage screening tool, whose primary aim is to identify with high certainty those who do not require further assessment, thereby concentrating diagnostic resources on the approximately one-quarter of the population flagged for follow-up. Further categorization into four risk levels also adds clinical refinement, allowing for various intervention strategies: instant referral to specialist for the Very High level (dyslexia rate of 69.4%), increased monitoring for High level (43.2%), and Normal educational support for Moderate and Low levels.

### 6.4 External validation and deployment roadmap

The present results derive from a single publicly available dataset. Before any clinical use, the framework requires (i) external validation on independent Dytective language cohorts, (ii) prospective, multi-center evaluation with device heterogeneity, and (iii) assessment of the regulatory and ethical requirements governing screening tools. We therefore present NEUROCOG-DysNet as a promising screening aid pending such validation rather than a deployment-ready clinical system.

### 6.5 Limitations

There are a few limitations that need to be highlighted. Firstly, DeLong's test for AUC comparison does not show statistical significance (*p* = 0.6946), and repeated-fold Wilcoxon and paired-*t* tests likewise indicate no significant AUC difference from the strongest baseline; the stacking ensemble's benefit therefore lies in a tunable sensitivity–specificity trade-off and well-calibrated probabilities rather than higher ranking performance. Secondly, a PPV of 0.3481 means that nearly two-thirds of the positive predictions are false positives, and this is an inevitable compromise to maintain a high sensitivity in a low-prevalence screening situation. Thirdly, the dataset comes from one platform only (Dytective) with predominantly Spanish-speaking participants, and generalization to other linguistic and cultural contexts remains to be established. Fourthly, the feature engineering pipeline, although systematic, is dependent on domain-informed but manually-designed transformations; feature learning through deep representations that are automated could uncover additional latent patterns that are not possible through handcrafted features.

## 7 Conclusion and future work

This paper introduces NEUROCOG-DysNet—a framework for feature enhancement at multiple scales of cognition with a stacking ensemble to help in more accurate dyslexia screening using gamified cognitive assessment data. It constructs 258 features in a hierarchical manner from five cognitive orders, then it selects only the 50 most relevant features using LightGBM importance, and finally, it combines seven diverse base classifiers by a stacking meta-learner to produce an AUC-ROC score of 0.8924 (95% CI: 0.8543–0.9253), sensitivity of 0.8077, and specificity of 0.8187. A cautious statistical assessment (DeLong *p* = 0.6946; McNemar *p* = 0.1443; repeated-fold Wilcoxon *p* = 0.695) shows that the stacking ensemble achieves AUC comparable to the best individual baseline while providing a favorable, tunable sensitivity–specificity balance; clinical utility measurement presents a 5.25 × screening lift and NPV of 0.9726.

Several promising directions will be the subject of future research. First, deep feature learning by means of autoencoders or graph neural networks on the task interaction structure may reveal latent cognitive patterns that are not accessible by handcrafted features. Second, longitudinal validation, which follows participants over time, would provide not only an opportunity to evaluate the framework's predictive power but also to detect cognitive profile changes due to development. Third, cross-linguistic validation of datasets from different orthographic systems (e.g., English, German, and Arabic) would be necessary to demonstrate the adaptability of the multi-scale cognitive feature hierarchy across languages. Further directions include multimodal integration (e.g., combining gamified interaction data with eye-tracking or reading-fluency signals), real-time on-device deployment, federated learning to enable privacy-preserving training across multiple institutions, and prospective clinical trials to establish real-world screening effectiveness.

## Data Availability

The Dytective dataset used in this study is publicly available on Kaggle (doi: 10.34740/kaggle/dsv/1617514). It was released under a Creative Commons license by Rello et al. (2020). The present study is a secondary analysis of de-identified, publicly available data and required no additional ethical approval.
